# Neutral sphingomyelinase-3 mediates TNF-stimulated oxidant activity in skeletal muscle

**DOI:** 10.1016/j.redox.2014.07.006

**Published:** 2014-07-30

**Authors:** Jennifer S. Moylan, Jeffrey D. Smith, Erin M. Wolf Horrell, Julie B. McLean, Gergana M. Deevska, Mark R. Bonnell, Mariana N. Nikolova-Karakashian, Michael B. Reid

**Affiliations:** a Department of Physiology, University of Kentucky, Lexington, KY, USA; b Center for Muscle Biology, University of Kentucky, Lexington, KY, USA; c Markey Cancer Center, University of Kentucky, Lexington, KY, USA; d Department of Surgery, University of Kentucky, Lexington, KY, USA

**Keywords:** Sphingolipid signaling, Neutral sphingomyelinase-3, nSMase3, Skeletal muscle, Free radicals, Reactive oxygen species, ROS, Oxidants, PAGE, polyacrylamide gel electrophoresis, VDAC, voltage dependent anion channel, KDEL, ER retention amino acid sequence.

## Abstract

**Aims:**

Sphingolipid and oxidant signaling affect glucose uptake, atrophy, and force production of skeletal muscle similarly and both are stimulated by tumor necrosis factor (TNF), suggesting a connection between systems. Sphingolipid signaling is initiated by neutral sphingomyelinase (nSMase), a family of agonist-activated effector enzymes. Northern blot analyses suggest that nSMase3 may be a striated muscle-specific nSMase. The present study tested the hypothesis that nSMase3 protein is expressed in skeletal muscle and functions to regulate TNF-stimulated oxidant production.

**Results:**

We demonstrate constitutive nSMase activity in skeletal muscles of healthy mice and humans and in differentiated C2C12 myotubes. nSMase3 (*Smpd4* gene) mRNA is highly expressed in muscle. An nSMase3 protein doublet (88 and 85 kD) is derived from alternative mRNA splicing of exon 11. The proteins partition differently. The full-length 88 kD isoform (nSMase3a) fractionates with membrane proteins that are resistant to detergent extraction; the 85 kD isoform lacking exon 11 (nSMase3b) is more readily extracted and fractionates with detergent soluble membrane proteins; neither variant is detected in the cytosol. By immunofluorescence microscopy, nSMase3 resides in both internal and sarcolemmal membranes. Finally, myotube nSMase activity and cytosolic oxidant activity are stimulated by TNF. Both if these responses are inhibited by nSMase3 knockdown.

**Innovation:**

These findings identify nSMase3 as an intermediate that links TNF receptor activation, sphingolipid signaling, and skeletal muscle oxidant production.

**Conclusion:**

Our data show that nSMase3 acts as a signaling nSMase in skeletal muscle that is essential for TNF-stimulated oxidant activity.

## Highlights

• First measures of endogenous nSMase3 protein in muscle.
• Detection of nSMase3 splice variant proteins.
• Identification of a functional role for nSMase3 in redox signaling.
• Identification of an intermediate in TNF/redox signaling.

## Graphical abstract

**Figure:**
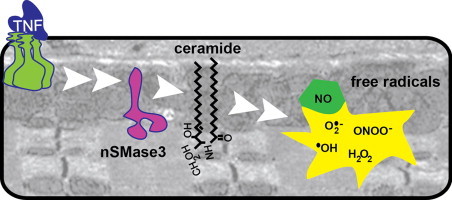

## Introduction

Membrane sphingolipids such as sphingomyelin play an important role in cell signaling. Hydrolysis of sphingomyelin leads to generation of sphingolipid signaling molecules, e.g. ceramide, sphingosine, sphingosine-1-phosphate. These effectors participate in pathways that regulate proliferation [46], apoptosis [26], cell cycle arrest [27], regeneration [2], glucose uptake [58], insulin resistance [48], viral uptake [3], immune response [4], vascular tone [60], mechanotransduction [12], aging [42], and memory [57]. Studies have shown that neutral sphingomyelinase 2 (nSMase2) is a predominant enzyme involved in these regulatory processes. nSMase2 hydrolyzes sphingomyelin in response to biological stimuli in liver, brain, immune cells, and other non-muscle cell types [9], [14], [29], [30], [44], [45], [55], [59].

Recently, sphingolipid signaling has been implicated in skeletal muscle functions like oxidant production [17], glucose uptake [1], [22], muscle cell adaptation [13], [24], [56], and force generation [17]. Exogenous nSMase causes a depression in diaphragm specific force that is suppressed by antioxidants [17]. In addition, nSMase activity may suppress myoblast differentiation [56] and stimulate muscle atrophy [13]. Correspondingly, nSMase activity is reduced after exercise [24].

Although it is clear that sphingolipid signaling plays a role in muscle function, nSMase2 appears to be of limited abundance in skeletal muscle [25]. Another nSMase, nSMase3, is reported to be highly expressed in heart and skeletal muscle [31]. However, the biochemistry of nSMase3 has not been widely studied. Overexpressed nSMase3 localizes to the endoplasmic reticulum (ER) and Golgi in breast (MCF-7) cancer cells [31]. Endogenous nSMase3 is found in mitochondrial-associated ER membranes in HeLa cells [8]. Overexpressed nSMase3 is activated by short (within 2 min) [31] but not long-term (14 h) [9] exposure to TNF. In addition, overexpression of nSMase3 enhances TNF- [8] and Adriamycin-induced apoptosis [11].

Our current study aimed to determine if nSMase3 contributes to redox signaling in skeletal muscle. Sphingolipid and oxidant signaling are stimulated by pro-inflammatory cytokines and seem to affect similar processes in skeletal muscle, e.g. glucose uptake [1], [7], [22], force production [17], [54], and fatigue [17], [51]. We therefore hypothesized that nSMase3 regulates TNF-stimulated oxidant production in skeletal muscle. We tested this hypothesis by addressing three questions: (1) Is nSMase3 expressed in skeletal muscle? (2) Does endogenous nSMase3 protein exhibit nSMase activity? (3) Is TNF-induced oxidant production mediated by nSMase3?

## Materials and methods

### Humans

Studies of human muscle were conducted at the University of Kentucky after approval by the Institutional Review Board. Diaphragm tissue biopsies were obtained from heart transplant organ donors after obtaining informed consent according to institutional guidelines.

### Animals

Studies were conducted at the University of Kentucky after approval by the Institutional Animal Care and Use Committee. Adult male C57BL/6J mice (6–8 week old, Harlan, Indianapolis, IN) were maintained by the Division of Laboratory Animal Resources in a 12:12 h dark:light cycle and received water and food ad libitum. Mice were deeply anesthetized by inhalation of isoflurane (Aerrane, Baxter Healthcare) and killed by exsanguination following cervical dislocation. Muscle tissues were harvested for enzyme activity assays and analysis of mRNA and protein.

### Myotubes

Myoblasts from the murine skeletal muscle-derived C2C12 cell line (American Type Culture Collection, Rockville, MD) were cultured as in Smith et al. [53]. Cells were seeded at 10,000 cells/cm^2^ in Dulbecco’s modified Eagle’s medium with 1.6 g/l sodium bicarbonate, 10% fetal bovine serum, and 100 U/ml PenStrep (Invitrogen, Carlsbad, CA) in 5% CO_2_ at 37 °C. After 2 days, myoblast cultures were serum restricted in FBS-free medium containing 2% horse serum. Mature myotubes were obtained 5 days after serum restriction. Medium was replaced every 48 h.

### nSMase activity

Cells (probe sonication, 5 s) or tissues (tissue homogenizer) were lysed in 25 mM Tris pH 7.2, 1 mM EDTA, phosphatase and protease inhibitor cocktails (Phosphatase Inhibitor Cocktails 2 and 3, Sigma-Aldrich, St. Louis, MO; Complete tablet, Roche Applied Science, Indianapolis, IN). Protein concentration was assessed using Coomassie Plus (Pierce-Thermo Scientific, Rockford, IL). We find that Triton X-100 obscures muscle nSMase activity. Accordingly, detergent was omitted from all isolation and assay buffers.

nSMase activity was assessed using two methods. The NBD-sphingomyelin method was used as described by Nikolova-Karakashian et al. [43] and Merrill et al. [37]. Sample (20 µg protein) was diluted into 30 µl assay buffer [25 mM Tris pH 7.5, 12 mM MgCl_2_, 15 µM fluorescent C6-NBD-sphingomyelin (6-((N-(7-nitrobenz-2-oxa-1,3-diazol-4-yl) amino) hexanoyl) sphingosyl phosphocholine); Invitrogen] and incubated at 37 °C for 3 h. All reaction buffers were supplemented with phosphatase, and protease inhibitor cocktails. Reactions were stopped by the addition of 500 µl methanol (HPLC grade, Mallinckrodt, St. Louis, MO). After further incubation at 37 °C for 30 min, the samples were centrifuged at 15,000*g* for 5 min and the generation of the fluorescent product, NBD-ceramide, was monitored by reverse phase HPLC using methanol:water:phosphoric acid (850:150:0.15, by volume) as a mobile phase [43].

The Amplex Red Sphingomyelinase Assay (Invitrogen, Carlsbad, CA) measures activity by converting nSMase-generated phosphocholine to choline with alkaline phosphatase, and choline to betaine and H_2_O_2_ with choline oxidase. H_2_O_2_ plus horseradish peroxidase act to oxidize non-fluorescent Amplex Red to fluorescent resorufin. The assay was performed according to the manufacturer’s specifications with minor modifications. Sample (20 µg protein) was diluted to 200 µl with assay mixture except that Triton was omitted. The mixture, ±sphingomyelin substrate, was incubated at 37 °C for 15–180 min and fluorescence emissions at 600 nm (530 nm excitation) were measured every 15–30 min by spectrofluorimeter (Synergy H1, Biotek, Winooski, VT). The first read was subtracted as baseline from subsequent reads. The increase in fluorescence caused by sphingomyelin supplementation reflected substrate-dependent phosphocholine release.

### PCR

PCR analyses were performed as described [19], [33]. RNA from myotubes or muscle tissue was isolated with TRIzol (Invitrogen). cDNA was synthesized using random hexamers or oligo dT and M-MLV reverse transcriptase (Promega, Madison, WI). Target mRNA was amplified using an Eppendorf Mastercycler (Hauppauge, NY) or quantified using the ABI 7500 Real, Time PCR System (Applied Biosystems-Invitrogen). Isoform specific primers are listed in Table 1. Primer efficiency was assessed by amplifying a 10-fold dilution series of target cDNA and plotting Ct values verses log sample concentration. The slope of the standard curve was used to calculate efficiency [efficiency=(10^(−1/slope)^−1)×100]. The abundance of target mRNA relative to rpl13A mRNA was determined using the comparative cycle threshold method [20], [36].

### Western blots

As described [53], myotubes or muscle tissues were homogenized in 2× protein loading buffer (120 mM Tris pH 7.5, 200 mM DTT, 20% glycerol, 4% SDS, 0.002% bromphenol blue). Equal amounts of protein were separated on 4–15% SDS-PAGE (Criterion, BioRad). Proteins were either stained using Simply Blue (Invitrogen) and scanned for total protein or transferred to PVDF membranes for western blot using the Odyssey Infrared Imaging System (LI-COR, Lincoln, NB). Fluorescence intensity data were normalized for total protein.

### Cell fractionation

Myotube fractions were enriched for cytosolic proteins, detergent-soluble proteins, and detergent-resistant proteins using the FOCUS Global Fractionation kit according to the manufacturer’s specifications with minor modifications (protocols A and C and G-Biosciences, St. Louis, MO). Specifically, the detergent insoluble pellet was resuspended in 2× protein loading buffer (see above). Concentrated (6×) protein loading buffer was added to the cytosolic and soluble protein fractions. Biological structures in the isolated fractions were assessed by western blot (Fig. 6) using antibodies for proteins of the cytosol (p38 MAPK, p38 mitogen-activated protein kinase), sarcolemma (TNFRI), endoplasmic reticulum (KDEL, ER targeting sequence), mitochondrial membrane (VDAC, voltage dependent anion channel), Golgi (giantin), and nucleus (lamin A/C).

### Antibodies

Primary antibodies from commercial sources were mouse anti-giantin, -VDAC (Abcam, Cambridge, MA), -ATP synthase α (Mitosciences, Abcam) rabbit anti-lamin A/C, -p38 MAPK (Cell Signaling Technologies, Danvers, MA); rabbit anti-nSMase3, -nSMase2, mouse anti-annexin 2 (ECM Biosciences, Versailles, KY); and rabbit anti-TNFR1 (Santa Cruz Biotechnology, Santa Cruz, CA). Secondary antibodies were as follows: donkey anti-mouse Alexa 680 (Molecular Probes-Invitrogen); goat anti-rabbit IRDye 800CW (LI-COR); donkey anti-rabbit DyLight 649; and donkey anti-mouse DyLight 549 (Jackson Immunoresearch, West Grove, PA).

### nSMase3 silencing

nSMase3 expression was silenced as described [41], [53] using morpholino (Gene Tools, Philomath, OR) and siRNA (Silencer Select, Ambion-Invitrogen) technologies. The morpholino spanned the start codon. Independent siRNAs targeted nSMase3 exons 11 and 18 (Table 1 and Fig. 6). Cultured cells were transfected 3 days after serum restriction using 10 µM morpholino plus Endo-Porter, or 20 nM siRNA plus Oligofectamine (Invitrogen). Myotubes were analyzed 48 h post-transfection.

### Native gel electrophoresis

Myotubes were lysed as for nSMase assay (above). Samples were diluted 1:1 with 2× native sample buffer (62.5 mM Tris, pH 6.8; 25% glycerol; 0.01% Bromphenol Blue) and electrophoresed through a 4–15% acrylamide gel (Criterion) using native running buffer (25 mM Tris, 192 mM glycine). Separated proteins were either transferred for western blotting (see above) or excised and incubated directly in 200 µl Amplex Red nSMase assay buffer for 3 h (see above).

### Immunofluorescence microscopy

Muscle cross-sections (6 µm) were fixed for 15 min at room temperature in 4% paraformaldehyde, permeabilized using 1% Triton X-100 for 15 min at 4 °C, and blocked for 2 h at room temperature using 1% normal serum [19], [40]. Prepared sections were probed overnight using antibodies to nSMase3 (1:100), ATP synthase-α (1:100), and annexin II (1:50). Secondary antibodies were incubated for 45 min (donkey anti-rabbit DyLight 649; donkey anti-mouse DyLight 594; 1:200). Sections were mounted with ProLong Gold antifade reagent with HEK293 (Invitrogen) and examined by fluorescence microscopy (LSM 5 Live Microscope, Zeiss, Thornwood, NY).

### GFP-nSMase3 overexpression

Primers containing XhoI and BamHI restriction sites and spanning the start and stop codons (Table 1) were used to PCR-amplify (Phusion High Fidelity Polymerase, New England Biolabs, Ipswich, MA) oligo dT-primed cDNA from myotubes. The amplified fragment was ligated into pAcGFP-C1 (Clontech, Mountain View, CA) to generate an N-terminal GFP-nSMase3 fusion protein. GFPnSMase3 plasmids were verified by DNA sequencing (Genewiz, South Plainfield, NJ). GFPnSMase3 or vector control was transiently transfected into cells 3 days after serum restriction using Lipofectamine LTX (Invitrogen). Myotubes were analyzed 72 h post-transfection and the data were normalized for transfection efficiency.

### Cytosolic oxidant activity

Live-cell oxidant activity was measured as described [17,23]. The fluorochrome probe 2′,7′-dichlorofluorescin diacetate (DCFH-DA 10 µM, Molecular Probes-Invitrogen) was loaded into myotubes 15 min prior to treatment with TNF (6 ng/ml, 20 min) or vehicle. Accumulation of the oxidized derivative DCF (excitation 480 nm, emission 520 nm) was measured using a fluorescence microscope (TE 2000S, Nikon, Melville, NY). Fluorescence images were captured using a CCD camera (CoolSNAP-ES, Roper Scientific Photometrics, Tucson, AZ) controlled by a computer with image acquisition software (NIS Elements, Nikon).

### Statistics

All comparisons were performed using Prism 5.0b (GraphPad Software, La Jolla, CA). We used 1-way ANOVA and Bonferroni’s test for multiple comparisons. All other comparisons were analyzed by *t*-test. Data are presented as mean±SE. Statistical significance was accepted when *P*<0.05.

## Results

### Neutral sphingomyelinase activity in skeletal muscle

**Fig 1:**
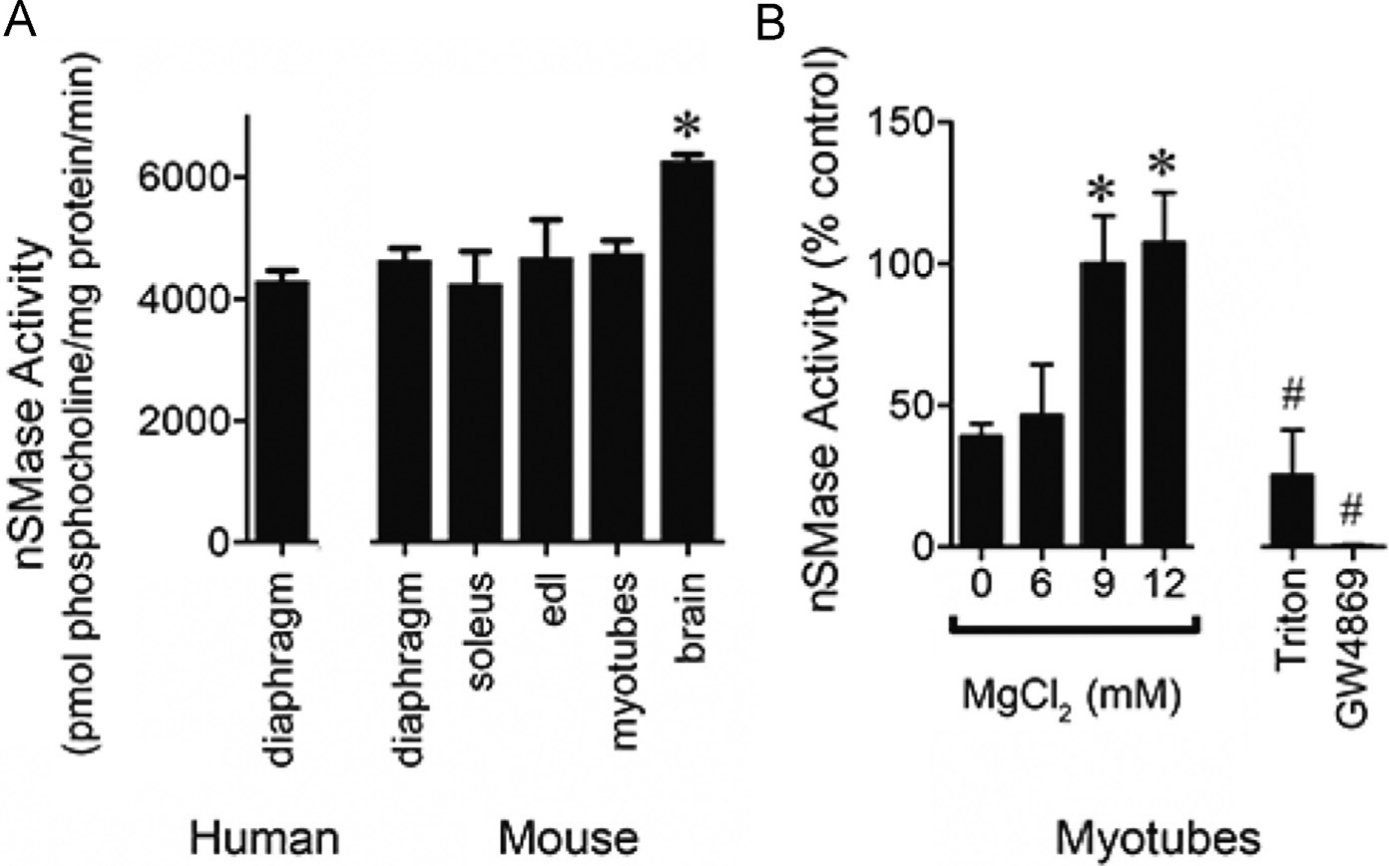
nSMase activity in skeletal muscle and myotubes. (A) nSMase activity in equal protein from tissues and myotubes assessed via choline release from sphingomyelin (*n*=3). Data are derived using a standard curve generated from serial dilutions of phosphocholine. *P*<0.02 by ANOVA, ^⁎^ different from human diaphragm and mouse soleus by Bonferroni’s multiple comparison. (B) Magnesium dependence of myotube nSMase activity (*n*=7) and sensitivity to Triton X-100 (0.1%, *n*=3) or GW4869 (10 µM, *n*=3) expressed as % control (MgCl_2_ 10 mM). *P*<0.001 by ANOVA, ^⁎^ different from 0 mM MgCl_2_, ^#^ different from control, by Bonferroni’s multiple comparison.

Equal amounts of protein from skeletal muscle, C2C12 myotube, or mouse brain homogenates were assessed for nSMase activity. nSMase activity was similar in all muscles evaluated (Fig. 1A). In comparison, the muscle activity is ~70% that of mouse brain (Fig. 1A). This is consistent with brain having abundant levels of nSMase2 [25]. In myotube extracts, activity at pH 7.5 exhibited characteristic Mg^2+^-dependence, with increasing activity between 6 and 12 mM MgCl_2_ (Fig. 1B). Neutral activity at 10 mM MgCl_2_ was depressed by Triton X-100 (0.1%) and abolished by 10 µM GW4869, a non-competitive nSMase inhibitor (Fig. 1B).

### Neutral sphingomyelinase mRNA in skeletal muscle

**Fig. 2:**
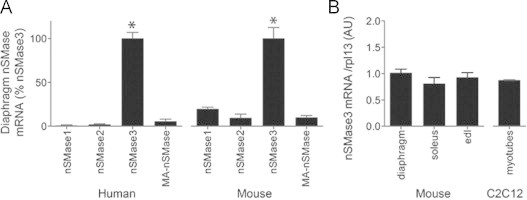
nSMase mRNA in skeletal muscle. (A) Real-time PCR of known nSMases in human and mouse diaphragm. (B) Real-time PCR of nSMase3 mRNA in different mouse skeletal muscles and myotubes (AU=arbitrary units, *n*=13 for human tissues, *n*=4 for mouse, *n*=3 for myotubes). Abundances are normalized to rpl13A. ^⁎^*P*<0.0001 by ANOVA.

In an effort to define the source(s) of nSMase activity in muscle, relative expression of nSMase mRNAs were compared. Quantitative real-time PCR analyses detected mRNA for all known nSMases (nSMase1, *Smpd2* gene; nSMase2, *Smpd3* gene; nSMase3, *Smpd4* gene; MA-nSMase, *Smpd5* gene); in human and mouse diaphragm (Fig. 2A). Primer efficiencies were similar (91–99% efficiency, data not shown) for each mRNA. In both human and mouse diaphragm, nSMase3 mRNA was most abundant, being present at levels 5–20 times greater than mRNA of other nSMases. nSMase3 mRNA was present at similar levels among mouse limb muscles, respiratory muscle, and myotubes (Fig. 2B). mRNA levels do not always mirror protein activity; however, these results suggest nSMase3 is an important source of nSMase activity in muscle.

### nSMase3 protein and splice variant expression in skeletal muscle

**Fig. 3:**
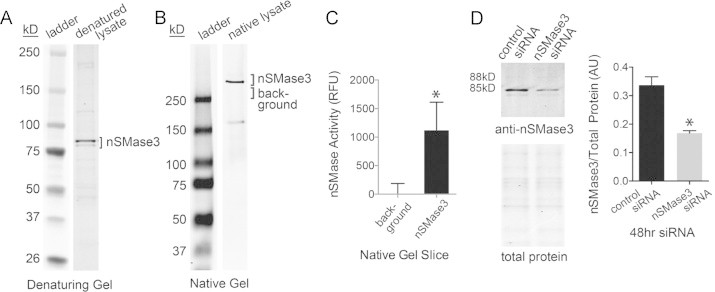
nSMase3 protein expression. (A) Western blot showing detection of a doublet using custom polyclonal nSMase3 antibodies. (B) Western blot under non-denaturing PAGE conditions shows migration of native nSMase3. (C) nSMase activity in non-denaturing gel slices of anti-nSMase3 reactive band compared to activity of non-specific gel slice (choline release, *n*=6), ^⁎^*P*<0.02. (D) siRNA specific for nSMase3 depletes the anti-nSMase3 reactive doublet. Sample volumes for western blot were adjusted for equal total protein, total protein was assessed by SDS-PAGE and simply blue staining (*n*=6, ^⁎^*P*<0.001).

nSMase3 protein was detected using custom polyclonal antibodies. In SDS-PAGE, nSMase3 antibody detected proteins of 88 and 85 kD (Fig. 3A). The most abundant protein appeared smaller than expected according to the predicted molecular weight of full-length mouse nSMase3 (93 kD). Membrane proteins commonly migrate faster than expected. Their inherent hydrophobicity lends to high SDS binding and a more compact structure that migrates faster than size predicts [50]. To confirm antibody specificity, myotube lysates were run on non-denaturing PAGE. Under these conditions a single band of ~290 kD was seen by western blot, indicating possible oligomerization (Fig. 3B). Excision of this band from the gel followed by activity assay [18] confirmed that the gel slice contains nSMase activity (Fig. 3C). In addition, myotubes were transfected with an nSMase3-specific siRNA (targeting exon 18 of the *Smpd4* gene). Both 88 and 85 kD proteins were depleted by siRNA (Fig. 3D). Quantification shows knockdown of nSMase3 protein was 50% effective (Fig. 3D). These results identify multiple isoforms and support a role for nSMase3 in skeletal muscle sphingolipid homeostasis.

**Fig. 4:**
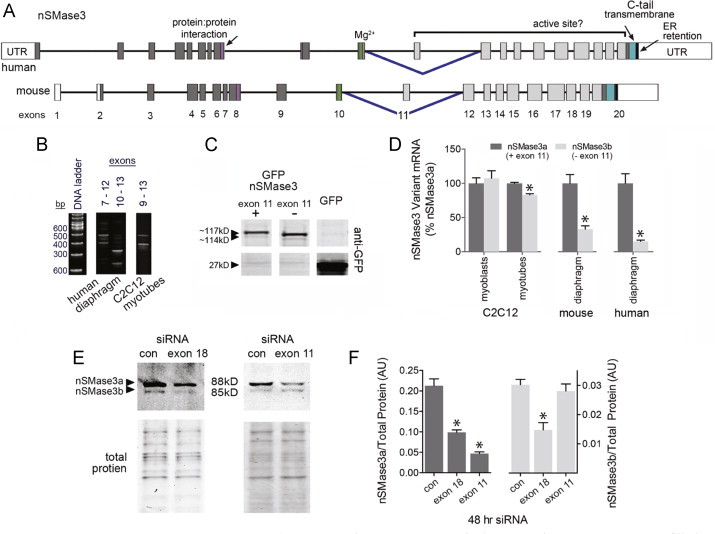
nSMase3 splice variant expression. (A) Structure of precursor mRNA for human and mouse nSMase3. Unfilled boxes represent untranslated regions (UTR); filled boxes are exons; lines are introns (scaled to 1/4 size relative to exons). Inverted blue carets indicate alternatively spliced exons. Below the maps are exon numbers. (B) Agarose gel shows human and myotube cDNA subjected to conventional PCR with primers that amplify select nSMase3 splice junctions (see Table 1). Numbers above the gels indicate the exon location for forward and reverse primers. Multiple bands indicate alternative splicing. (C) Western blot with anti-GFP shows expression of nSMase3 splice variant GFP-fusion clones. (D) Real-time PCR of nSMase3 splice variants. A forward primer in exon 11 and a reverse primer in exon 12 detects nSMase3a; a forward primer that spans the exon 10–exon 12 splice junction and a reverse primer in exon 12 detects nSMase3b (see Table 1; *n*=6 for human tissues, *n*=4 for mouse, *n*=3 for myoblasts or myotubes), ^⁎^*P*<0.05 vs. nSMase3a. (E) Western blot and total protein of extracts from siRNA treated myotubes, siRNAs (20 nM) targeted nSMase3a and b (exon 18) or nSMase3a (exon 11). siRNA primer sequences are listed in Table 1. (F) Quantification of E (*n*=9 control siRNA, *n*=6 exon 18 siRNA, *n*=3 exon 11 siRNA), ^⁎^*P*<0.05 vs. control.

The nSMase3 precursor mRNA structure and it is homology between human and mouse is shown in Fig. 4A. It has a unique protein:protein interaction domain and a C-terminal transmembrane domain that is juxtaposed to the putative active site. In addition, nSMase3 has a potential N-linked glycosylation site in exon 17 of the c-terminus (not shown). We tested whether the 88 and 85 kD isoforms are the result of glycosylation. However, treatment with N-glycosidase F did not alter migration (data not shown).

Next we tested whether the protein isoforms were derived from alternative splicing. nSMase3 appears to undergoes complex RNA processing. NCBI Gene (http://www.ncbi.nlm.nih.gov/gene) and Aceview list multiple (4 and 17, respectively) coding nSMase3 mRNA sequences (https://www.ncbi.nlm.nih.gov/IEB/Research/Acembly/). Conventional PCR, cDNA cloning and sequencing, and real-time PCR demonstrated that mouse skeletal muscle expresses at least two splice variants (mouse NM_029945.3, NM_001164610.1; human NM_017951.4, NM_017751.4). Fig. 4B depicts an agarose gel containing conventional PCR products from amplification of select nSMase3 exons. The labels above each lane indicate the exons that were amplified. Multiple bands indicate potential splice variants. A potential 100 bp deletion is shown between exons 7–12 and 10–13 in human diaphragm and between exons 9 and 13 in mouse myotubes. These results suggest a conserved splice variant is expressed in mouse and human skeletal muscle. Amplification and cloning of nSMase3 full-length cDNA resulted in recovery of two unique cDNA clones fused with an N-terminal GFP (Fig. 4C). Consistent with the conventional PCR results, DNA sequencing revealed that the larger clone contains all 20 exons of the nSMase3 coding region (mouse NM_029945.3) while the smaller clone lacks the 100 bp exon 11 (mouse NM_001164610.1). We have designated these splice variants nSMase3a and nSMase3b, respectively. Primers were designed to specifically detect nSMase3a and b mRNA, i.e., with or without exon 11. Using these, we confirmed that both nSMase3a and nSMase3b mRNAs are constitutively expressed in muscle. The data are shown in Fig. 4D. Human muscle, mouse muscle, and myotubes express more nSMase3a than b whereas myoblasts express the variants at equal levels.

The predicted molecular weights of nSMase3a and b correspond to the 88 and 85 kD isoforms recognized by the nSMase3 antibody (Fig. 3). To confirm the identity of these proteins, myotubes were transfected with siRNA targeting nSMase3a and b (exon 18) or nSMase3a only (exon 11). Western blot of extracts from transfected myotubes showed that exon 18 siRNA depleted both isoforms, while exon 11 siRNA depleted only the 88 kD protein (Fig. 4E and F). These results indicate the 88 kD protein is nSMase3a and the 85 kD protein is nSMase3b. In agreement with the mRNA data, nSMase3a is more abundant than nSMase3b.

### nSMase3 distribution

**Fig 5:**
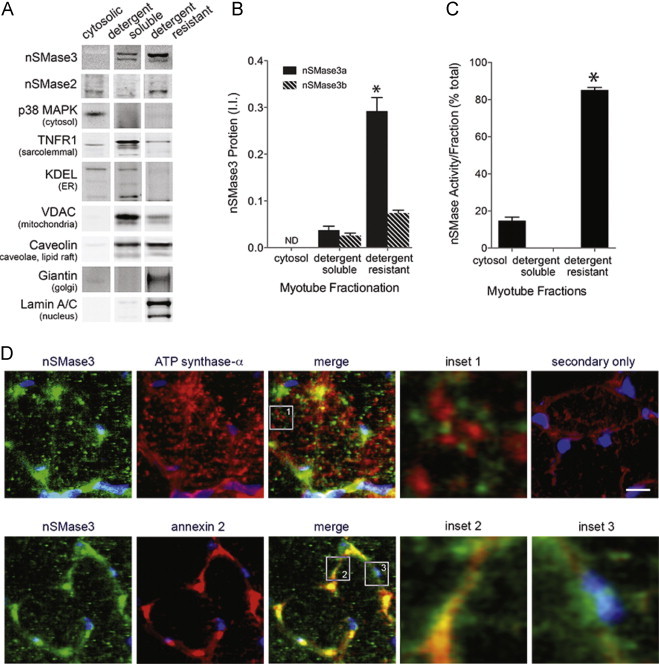
Distribution of nSMase3 in cellular fractions. (A) Western blot of myotube fractions. Antibodies to proteins that reside in discreet compartments show the composition of fractions. (B) Quantification of nSMase3 protein detected in A (*n*=6, ^⁎^*P*<0.0001 vs. detergent-soluble fraction and nSMase3b). (C) nSMase activity per fraction (choline release, *n*=3, ^⁎^*P*<0.0001 vs. cytosol). (D) Upper panel: immunofluorescent staining of mouse diaphragm sections with anti-nSMase3 (green) and anti-ATP synthase-α (red). Nuclei are stained with DAPI (blue). Inset 1 shows location relative to inner mitochondrial membrane. Lower panel: co-staining with nSMase3 (green) and annexin 2 (red) antibodies. Inset 2 shows nSMase3 staining near the plasma membrane, inset 3 shows perinuclear staining. Upper right panel, secondary antibodies only. Scale bar=10 µm.

Fig. 5A demonstrates that nSMase3a and b distribute differently between cellular fractions. The cytosolic fraction contained the majority of total myotube protein (84±0.7% SEM) and cytosolic p38 MAPK but no detectable levels of nSMase3. A portion of nSMase2 was found in the cytosol. The detergent-soluble fraction contained 9% (±0.1 SEM) of total protein and membrane proteins commonly associated with sarcolemma (TNF receptor 1, TNFR1), mitochondria (voltage dependent anion channel, VDAC), endoplasmic reticulum (ER retention amino acid sequence, KDEL), or caveolae (caveolin). Approximately 12% of total nSMase3 protein composed of equal amounts of nSMase3a and b were found in the soluble fraction. The detergent-resistant fraction, which contained 6% (±0.1 SEM) of total protein, is reported to have proteins concentrated in caveolin-, cholesterol-, and sphingolipid-rich membranes [46], [47], [48]. These membrane regions are considered hot spots for receptor-mediated signaling [35]. Correspondingly, we find a small proportion of TNFRI in the detergent resistant fraction and a majority of caveolin. It is also enriched with proteins of the Golgi (giantin) and nucleus (lamin A/C) (Fig. 5A). This fraction contained the majority (88%) of total nSMase3 and was composed primarily of nSMase3a. These data suggest that nSMase3a and b may serve different functions in myotube sphingolipid metabolism.

The distribution of nSMase activity per fraction is consistent with nSMase3 protein localization. The majority of activity is in the detergent-resistant fraction (82±1.8%, *P*<0.0001). A minor portion (18%) is cytosolic and may be attributed to cytosolic nSMase2. Detergent-soluble activity was undetectable, likely due to Triton in the sample.

Immunofluorescence staining also demonstrated discrete cellular localization. nSMase3 antibody stained mouse diaphragm in a punctate pattern within and on the periphery of the myofiber (Fig. 4D). Interior nSMase3 staining did not co-localize with inner mitochondrial membrane structures as revealed by co-staining with ATP synthase-α (Fig. 4D, upper panel and inset 1). Perimeter staining near the plasma membrane overlapped with staining for annexin 2, a cytoskeletal protein that localizes to the inner and outer plasma membrane (Fig. 4D, lower panel and inset 2). nSMase3 staining also surrounded some nuclei (Fig. 4D, lower panel and inset 3, blue DAPI stain). These data support prior reports that nSMase3 does not localize to the mitochondria but rather to mitochondrial-associated ER membranes [8], [11], [31] and also suggest some sarcolemmal and perinuclear localization.

### nSMase3 activity

**Fig. 6:**
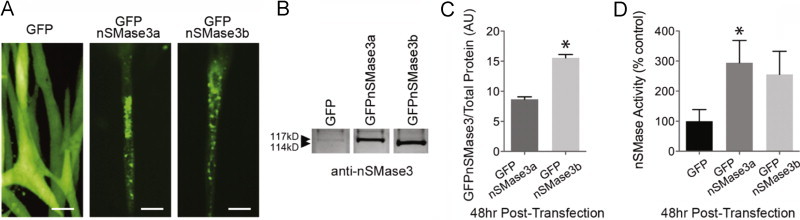
nSMase3 splice variant activity. (A) Fluorescence images of GFPnSMase3a and GFPnSMase3b in myotubes (scale bar=100 µm). (B) Western blot of lysates transfected with GFPnSMase3a or b using anti-nSMase3. (C) Quantification of B (*n*=3), *P*<0.001 vs. GFPnSMase3a. (D) nSMase activity in lysates from myotubes transfected with GFPnSMase3a or GFPnSMase3b (choline release, *n*=6), ^⁎^*P*<0.05 vs. GFP control.

GFPnSMase3 variants expressed in myotubes localized in punctate clusters (Fig. 6A) similar to staining patterns of the endogenous protein in mouse diaphragm (Fig. 5D). Western blot using the nSMase3 antibody confirms GFPnSMase3 proteins are overexpressed relative to endogenous nSMase3 (Fig. 6B). GFPnSMase3a is functionally active. This is shown in Fig. 6D where overexpression of nSMase3a led to elevated nSMase activity. Overexpression of nSMase3b tended to increase activity but this increase was not statistically significant. Quantification of GFPnSMase3 overexpression (Fig. 6C) showed that overexpression of GFPnSMase3b was greater than GFPnSMase3a (180±6%, *P*<0.001), but activity was slightly lower (Fig. 6C). Thus there may be differences in enzymatic properties of the variants.

### nSMase3 and receptor-mediated signaling

**Fig. 7:**
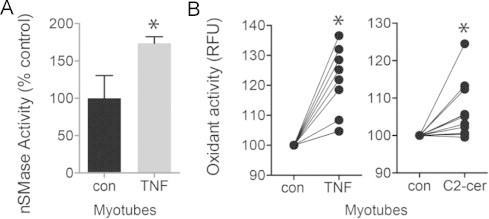
TNF, sphingomyelin, and oxidant signaling. (A) TNF-induced nSMase activity in myotubes, 5 min pretreatment of TNF (6 ng/ml, choline release, *n*=9), ^⁎^*P*<0.05. (B) Increased oxidant activity in C2C12 myotubes pretreated for 20 min with TNF (6 ng/ml) or C2-ceramide (10 µM). Measured by DCF fluorescence assay, each treatment was applied to 12-wells of cultured myotubes, the 5 brightest myotubes per well were analyzed, each data point is an average of these 5 myotubes (RFU=relative fluorescence units), ^⁎^*P*<0.02 vs. untreated, paired comparison.

**Fig. 8:**
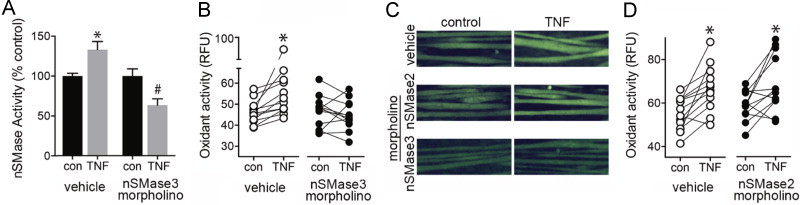
TNF, nSMase3, and oxidant signaling. (A) nSMase activity in myotubes transfected with vehicle (Endo-Porter) or nSMase3 morpholino (10 µM)±TNF (6 ng/ml, 15 min, *n*=6, choline release assay, *P*<0.0001 by ANOVA, ^⁎^ different from control, ^#^ different from TNF-treated vehicle, by Bonferroni’s multiple comparison). (B) Quantification oxidant activity from myotubes transfected with vehicle (Endo-Porter) or nSMase3 morpholino (10 µM)±TNF (6 ng/ml, 20 min). (C) Representative images of myotubes transfected with vehicle (Endo-Porter), nSMase2, or nSMase3 morpholino±TNF. (D) Quantification of oxidant activity from myotubes transfected with vehicle (Endo-Porter) or nSMase2 morpholino (10 µM)±TNF. Oxidant activity analysis was performed as in Fig. 7B.

**Fig. 9:**
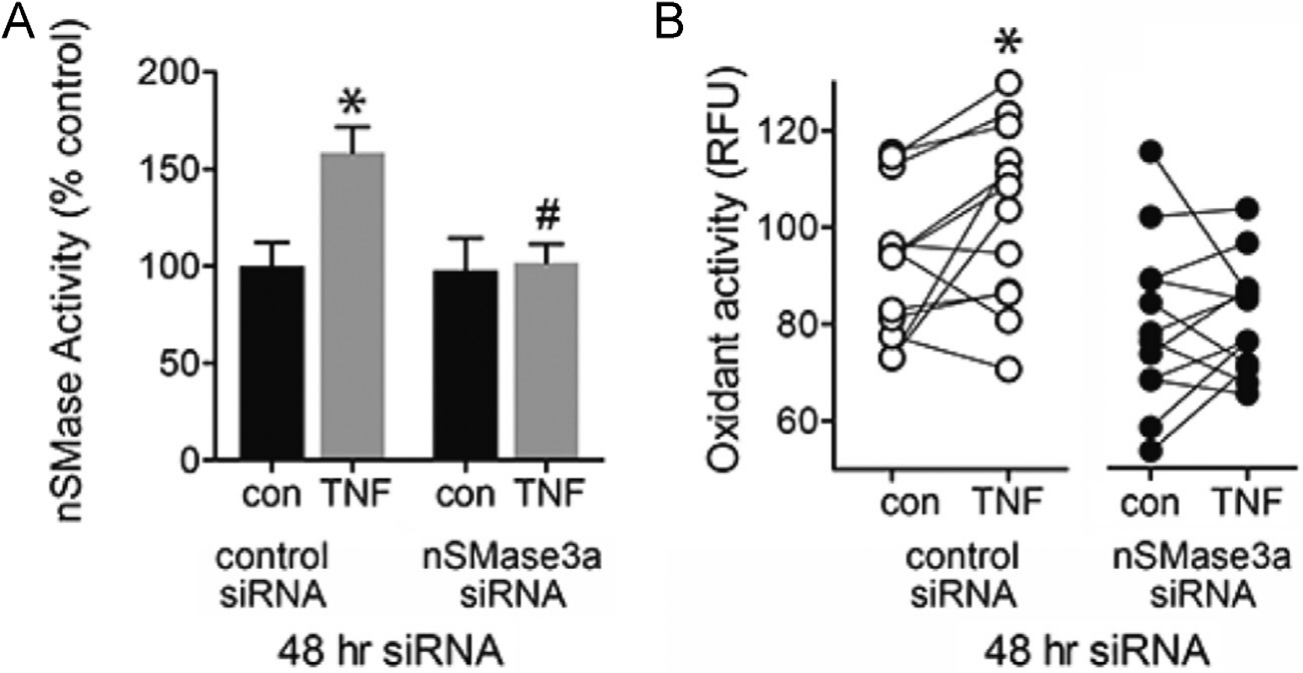
nSMase3a and oxidant signaling. (A) nSMase activity in myotubes transfected with control or siRNA to nSMase3a (exon 11, 20 nM)±TNF (6 ng/ml, 5 min, *n*=4, ceramide release assay, *P*<0.002 by ANOVA, ^⁎^ different from untreated, ^#^different from TNF-treated control siRNA by Bonferroni’s multiple comparison). (B) Oxidant activity in myotubes transfected with control or siRNA to nSMase3a (as in A)±TNF (6 ng/ml, 20 min, *n*=9 per group, ^⁎^*P*<0.001, paired comparisons). Oxidant activity analysis was performed as in Fig. 7B.

TNF stimulates cytosolic oxidant activity in skeletal muscle, a response that is detectable within minutes and is mediated via TNF receptor subtype 1 [23], [34]. Similarly, Ferreira et al. have shown that direct exposure to bacterial SMase or exogenous ceramide also increases oxidant activity [17]. These reports implicate signaling lipids as receptor-activated regulators of skeletal muscle oxidant production. Our current data suggests this process is regulated by nSMase3. Data in Fig. 7, Fig. 8, Fig. 9 support this idea. Fig. 7 describes TNF-stimulated nSMase activity (Fig. 7A) and confirms that TNF or C2-ceramide increase oxidant activity (Fig. 7B). Suppression of nSMase3 by morpholino blocked TNF-stimulated nSMase activity (Fig. 8A) and oxidant production (Fig. 8D). Fig. 8C shows representative fluorescence images of control and TNF-treated myotubes transfected with vehicle (Endo-Porter), nSMase2 morpholino, or nSMase3 morpholino. Vehicle or nSMase2 morpholino controls demonstrate that depression of oxidant activity is specific for the nSMase3 morpholino (Fig. 8D).

To confirm the morpholino results, myotubes were transfected with nSMase3a siRNA (exon 11). TNF stimulated both nSMase activity (Fig. 9A) and oxidant production (Fig. 9B) in control myotubes but failed to do so in myotubes transfected with nSMase3a siRNA. We were unable to determine if nSMase3b contributes to oxidant signaling because an siRNA designed to target design an nSMase3b specific siRNA. However, siRNA knocking down both nSMase3a and 3b (exon 18) reduced oxidant production to the same degree as nSMase3a siRNA alone (data not shown), suggesting nSMase3a is sufficient to mediate TNF-stimulated oxidant production.

## Discussion

Muscle nSMase activity has been detected in rabbit hind limb muscles [18], rat soleus and gastrocnemius [15], and human vastus lateralis [24]. In this report, we compared nSMase activity in mouse brain, diaphragm, extensor digitorum longus (EDL), soleus, and human diaphragm. We found that mouse muscle exhibits significant nSMase activity similar to that of human diaphragm and 70% that of mouse brain. Muscle SMase activity has characteristics that are typically observed for a magnesium-dependent nSMase. The importance of nSMase in regulating cell function, especially cellular stress responses, has long been known. Recent research has shown that nSMases are a family of proteins with similar enzyme activity, but with seemingly distinct function in sphingolipid homeostasis and signaling. The nSMase3 isoform was cloned in 2006 [31]; this report suggested that nSMase3 was preferentially expressed in muscle. Our data indicate that nSMase3 contributes to the overall nSMase activity in muscle. The supporting evidence is as follows: (i) comparison of all four nSMase isoforms in muscle shows that nSMase3 mRNA is abundantly expressed; (ii) overexpression of nSMase3 in myotubes elevates overall nSMase activity; (iii) depletion of nSMase3 using siRNA decreases nSMase activity.

In skeletal muscle, sphingolipids appear to be important mediators of TNF signaling. For example, the response of murine muscle to TNF, i.e., increased oxidant production, is mimicked by direct exposure to either C6-ceramide or bacterial Smase [17], [23]. Our current experiments demonstrate that TNF stimulates endogenous nSMase activity. We attribute some of this to nSMase3 since nSMase3 silencing prevents the rise in activity. Finally we show that nSMase3 is essential for the rise in cytosolic oxidant activity stimulated by TNF. By two independent methods, nSMase3 morpholino or nSMase3 siRNA, we show that depletion of nSMase3 abolishes the increase in oxidants after TNF exposure. In aggregate, these findings provide evidence for a TNF–nSMase3-oxidant pathway.

Instead of nSMase3, the nSMase2 isoform has been shown to mediate TNF action in non-muscle cell types [9]. Our data do not exclude the possibility that nSMase2 mediates other TNF functions in muscle. The two enzymes vary in their predicted structures and subcellular localizations, suggesting different roles in signaling and distinct mechanisms of regulation. In particular, nSMase2 has two transmembrane domains and N-palmitoylation is required for membrane attachment [59]. Regulated transport of nSMase2 to the plasma membrane is required for signaling [10], [38]. In contrast, nSMase3 is a c-tail-anchored membrane protein. Such proteins localize to target membranes after full emergence and release from the ribosome into the cytosol [review [6]]. Tail-anchored proteins localize either unassisted or by chaperone to structures that include ER, outer mitochondrial, and peroxisomal membranes. From the ER, these proteins can be transported to the Golgi and plasma membrane.

Our data lend support to previous studies that report multiple locations for nSMase3. Using cell fractionation and immunofluorescence staining of the endogenous protein, we found sites near the plasma membrane, perinuclear region, and areas surrounding mitochondria. A peri-mitochondrial relationship was also reported by Chipuk et al. [8]. They found endogenous nSMase3 in mitochondrial-associated ER membranes from mouse liver. Others have found GFP-tagged nSMase3 in ER and Golgi membranes [8], [11], [31]. The variety of reported locations may reflect the large number of potential nSMase3 isoforms predicted by Aceview and NCBI Gene. Alternatively, the apparent ubiquity in membrane sites could indicate an important role for nSMase3 in sphingolipid homeostasis of cellular membranes.

nSMase-generated ceramide species are not thought to travel from their site of production. Thus the location of nSMase3 could provide clues as to the source of oxidant production. For example, ceramide generated at the plasma membrane might alter membrane properties to allow for assembly and clustering of NADPH oxidase subunits [28] or might allow calcium influx and nNOS activation [16]. Alternatively, ER-localized nSMase3 might activate intracellular ER-associated NADPH oxidase [21]. A growing body of evidence points to mitochondria as a major source of reactive oxygen species in skeletal muscle [32], [39], [49]. Thus, nSMase3 found in mitochondria-associated ER membranes [8] might alter membrane properties to stimulate mitochondrial oxidant production.

Finally, our study confirms the expression of alternative nSMase3 transcripts. Multiple validated splice variants for nSMase3 are reported in NCBI Gene. We find that at least two of these, designated nSMase3a and nSMase3b, are constitutively expressed in skeletal muscle and appear to distribute differently. nSMase3a fractionates with proteins of cholesterol-rich (caveolin), and Golgi (giantin) membranes. nSMase3b distributes more equally and co-fractionates with mitochondrial (VDAC), ER (KDEL), and plasma membrane (TNFRI) proteins. Different localization patterns suggest that nSMase3a and nSMase3b may serve separate functions, respond to different stimuli, or hydrolyze distinct sphingomyelin pools. These finding indicate that nSMase3 function may depend upon epigenetic mechanisms of regulation that have not yet been identified.

## Conclusions

In summary, our data demonstrate nSMase activity in skeletal muscles of humans and mice. We show that nSMase3 mRNA is highly expressed in skeletal muscle. We suggest that sphingolipid signaling in skeletal muscle is derived in part from nSMase3 and that nSMase3 regulates skeletal muscle oxidant production. Oxidants are important mediators of TNF effects in skeletal muscle, modulating force production [23], [52] and muscle quality [5]. Prior to our study, signaling intermediates between TNF-receptor binding and oxidant production had not been identified. We identify nSMase3 as an important mediator of TNF/redox signaling that may prove critical for redox-sensitive aspects of muscle function.

## Conflict of interest

No competing financial interests exist.

## Figures and Tables

**Table 1:** Oligonucleotide primers.

Real time PCR	Forward (5′–3′)	Reverse (5′–3′)
**Mouse**		
nSMase1	CTCGCCGCCCTTGCT	CAGCCGTAGAGAAAAGTTGAGCTT
nSmase2	CACCAACACCTCCATCAGTG	GTCAGCCTGTTCTCCAGAGG
nSMase3	CCCTACGCACAGACCTTGTCA	AAGACCTTAGCCACTCGGAAGA
nSMase3a	ACCGACTCACTGTCTCCAGC	GACTCTTGGTAGGCGTGGAG
nSMase3b	CTCATGCCAAGGAGTCCTTC	AAGGCATGCAGGTGCTTC
nSMase-MT	TCATGGGAGTTACCCAGCTC	CGACATTCATCCCCTTGTCT
rpl13A	GCAAGTTCACAGAGGTCCTCAA	GGCATGAGGCAAACAGTCTTTA

**Human**		
nSMase1	TTTGGTGTCCGCATTGACTA	TAGAGCTGGGGTTCTGCTGT
nSMase2	GGAAGGCCGAGGTGGAA	CCCCCGAAGACACCATCA
nSMase3	CACCCAGGATGAGAATGGAAA	GTCCGTCCTCACCCACGAT
nSMase-MT	GAGCTAAGCCCTGGAGAGGT	GACACTGAGACTCGGAGCTG
rpl13A	TCCTGGTCTGAGCCCAATAAA	CAGGGCAACAATGGAGGAA

**Conventional PCR**	**Forward (5**′–**3**′**)**	**Reverse (5**′–**3**′**)**
**Human nSMase3**		
Exons 7–12	TCTTTGCCTTGAGCCTCATC	ACCACCAACACATGCTCCTC
Exons 10–13	TGTTGAAATGTGGCTTCATCA	GAGTTTCTGCTGGACGAACC

**Mouse nSMase3**		
Exons 9–13	TTTGTGGAAATGTGGCTTCA	CCTGTTTCTCAGGGGCATAC

**Cloning primers**	**Forward (5**′–**3**′**)**	**Reverse (5**′–**3**′**)**
		
	ccgctcgagCTATGGCGTTCCCTCAC	cgggatccTCAGAGCTGGTGCAGCTT

**Morpholino oligos**	**Antisense**	
nSMase2	ACAAAACCATTGCAGCTCATGGGC	
nSMase3	TGAGGGAACGCCATAGCAGACTCCC	

**siRNA oligos**	**Sense**	**Antisense**
nSmase3 exon 11	GAGGUUCUACACUACCGACtt	GUCGGUAGUGUAGAACCUCca
nSmase3 exon 18	GAUCAUUAAUGGUCUGCGAtt	UCGCAGACCAUUAAUGAUCtg

## References

[1] Adams J.M., Pratipanawatr T., Berria R., Wang E., DeFronzo R.A., Sullards M.C., Mandarino L.J. (2004). Ceramide content is increased in skeletal muscle from obese insulin-resistant humans. Diabetes.

[2] Alessenko A., Chatterjee S. (1995). Neutral sphingomyelinase: localization in rat liver nuclei and involvement in regeneration/proliferation. Molecular and Cellular Biochemistry.

[3] Avota E., Gulbins E., Schneider-Schaulies S. (2011). DC-SIGN mediated sphingomyelinase-activation and ceramide generation is essential for enhancement of viral uptake in dendritic cells. PLoS Pathogens.

[4] Ballou L.R., Laulederkind S.J., Rosloniec E.F., Raghow R. (1996). Ceramide signalling and the immune response. Biochimica et Biophysica Acta.

[5] Barker T., Traber M.G. (2007). From animals to humans: evidence linking oxidative stress as a causative factor in muscle atrophy. Journal of Physiology.

[6] Borgese N., Fasana E. (2011). Targeting pathways of C-tail-anchored proteins. Biochimica et Biophysica Acta.

[7] Chambers M.A., Moylan J.S., Smith J.D., Goodyear L.J., Reid M.B. (2009). Stretch-stimulated glucose uptake in skeletal muscle is mediated by reactive oxygen species and p38 MAP-kinase. Journal of Physiology.

[8] Chipuk J.E., McStay G.P., Bharti A., Kuwana T., Clarke C.J., Siskind L.J., Obeid L.M., Green D.R. (2012). Sphingolipid metabolism cooperates with BAK and BAX to promote the mitochondrial pathway of apoptosis. Cell.

[9] Clarke C.J., Cloessner E.A., Roddy P.L., Hannun Y.A. (2011). Neutral sphingomyelinase 2 (nSMase2) is the primary neutral sphingomyelinase isoform activated by tumour necrosis factor-alpha in MCF-7 cells. Biochemical Journal.

[10] Clarke C.J., Guthrie J.M., Hannun Y.A. (2008). Regulation of neutral sphingomyelinase-2 (nSMase2) by tumor necrosis factor-alpha involves protein kinase C-delta in lung epithelial cells. Molecular Pharmacology.

[11] Corcoran C.A., He Q., Ponnusamy S., Ogretmen B., Huang Y., Sheikh M.S. (2008). Neutral sphingomyelinase-3 is a DNA damage and nongenotoxic stress-regulated gene that is deregulated in human malignancies. Molecular Cancer Research.

[12] Czarny M., Schnitzer J.E. (2004). Neutral sphingomyelinase inhibitor scyphostatin prevents and ceramide mimics mechanotransduction in vascular endothelium. American Journal of Physiology: Heart and Circulatory Physiology.

[13] De Larichaudy J., Zufferli A., Serra F., Isidori A.M., Naro F., Dessalle K., Desgeorges M., Piraud M., Cheillan D., Vidal H., Lefai E., Némoz G. (2012). TNF-α- and tumor-induced skeletal muscle atrophy involves sphingolipid metabolism. Skeletal Muscle.

[14] Dobierzewska A., Giltiay N.V., Sabapathi S., Karakashian A.A., Nikolova-Karakashian M.N. (2011). Protein phosphatase 2A and neutral sphingomyelinase 2 regulate IRAK-1 protein ubiquitination and degradation in response to interleukin-1beta. Journal of Biological Chemistry.

[15] Dobrzyń A., Górski J. (2002). Ceramides and sphingomyelins in skeletal muscles of the rat: Content and composition. Effect of prolonged exercise. American Journal of Physiology: Endocrinology and Metabolism.

[16] Eugenin E.A., King J.E., Nath A., Calderon T.M., Zukin R.S., Bennett M.V., Berman J.W. (2007). HIV-tat induces formation of an LRP-PSD-95- NMDAR-nNOS complex that promotes apoptosis in neurons and astrocytes. Proceedings of the National Academy of Sciences of the United States of America.

[17] Ferreira L.F., Moylan J.S., Gilliam L.A., Smith J.D., Nikolova-Karakashian M., Reid M.B. (2010). Sphingomyelinase stimulates oxidant signaling to weaken skeletal muscle and promote fatigue. American Journal of Physiology: Cell Physiology.

[18] Ghosh N., Sabbadini R., Chatterjee S. (1998). Identification, partial purification, and localization of a neutral sphingomyelinase in rabbit skeletal muscle: neutral sphingomyelinase in skeletal muscle. Molecular and Cellular Biochemistry.

[19] Gilliam L.A., Moylan J.S., Ferreira L.F., Reid M.B. (2011). TNF/TNFR1 signaling mediates doxorubicin-induced diaphragm weakness. American Journal of Physiology: Lung Cellular and Molecular Physiology.

[20] Giulietti A., Overbergh L., Valckx D., Decallonne B., Bouillon R., Mathieu C. (2001). An overview of real-time quantitative PCR: applications to quantify cytokine gene expression. Methods.

[21] Glass M.J., Huang J., Oselkin M., Tarsitano M.J., Wang G., Iadecola C., Pickel V.M. (2006). Subcellular localization of nicotinamide adenine dinucleotide phosphate oxidase subunits in neurons and astroglia of the rat medial nucleus tractus solitarius: relationship with tyrosine hydroxylase immunoreactive neurons. Neuroscience.

[22] Górska M., Dobrzyń A., Zendzian-Piotrowska M., Górski J. (2004). Effect of streptozotocin-diabetes on the functioning of the sphingomyelin-signalling pathway in skeletal muscles of the rat. Hormone and Metabolic Research.

[23] Hardin B.J., Campbell K.S., Smith J.D., Arbogast S., Smith J., Moylan J.S., Reid M.B. (2008). TNF-alpha acts via TNFR1 and muscle-derived oxidants to depress myofibrillar force in murine skeletal muscle. Journal of Applied Physiology.

[24] Helge J.W., Dobrzyn A., Saltin B., Gorski J. (2004). Exercise and training effects on ceramide metabolism in human skeletal muscle. Experimental Physiology.

[25] Hofmann K., Tomiuk S., Wolff G., Stoffel W. (2000). Cloning and characterization of the mammalian brain-specific, Mg^2+^-dependent neutral sphingomyelinase. Proceedings of the National Academy of Sciences of the United States of America.

[26] Jarvis W.D., Kolesnick R.N., Fornari F.A., Traylor R.S., Gewirtz D.A., Grant S. (1994). Induction of apoptotic DNA damage and cell death by activation of the sphingomyelin pathway. Proceedings of the National Academy of Sciences of the United States of America.

[27] Jayadev S., Liu B., Bielawska A.E., Lee J.Y., Nazaire F., Pushkareva MYu, Obeid L.M., Hannun Y.A. (1995). Role for ceramide in cell cycle arrest. Journal of Biological Chemistry.

[28] Jin S., Zhou F., Katirai F., Li P.L. (2011). Lipid raft redox signaling: molecular mechanisms in health and disease. Antioxidants & Redox Signaling.

[29] Khavandgar Z., Poirier C., Clarke C.J., Li J., Wang N., McKee M.D., Hannun Y.A., Murshed M. (2011). A cell-autonomous requirement for neutral sphingomyelinase 2 in bone mineralization. Journal of Cell Biology.

[30] Kolesnick R.N. (1989). Sphingomyelinase action inhibits phorbol ester-induced differentiation of human promyelocytic leukemic (HL-60) cells. Journal of Biological Chemistry.

[31] Krut O., Wiegmann K., Kashkar H., Yazdanpanah B., Krönke M. (2006). Novel tumor necrosis factor-responsive mammalian neutral sphingomyelinase-3 is a C-tail-anchored protein. Journal of Biological Chemistry.

[32] Lee H.Y., Kaneki M., Andreas J., Tompkins R.G., Martyn J.A. (2011). Novel mitochondria-targeted antioxidant peptide ameliorates burn-induced apoptosis and endoplasmic reticulum stress in the skeletal muscle of mice. Shock.

[33] Li W., Moylan J.S., Chambers M.A., Smith J., Reid M.B. (2009). Interleukin-1 stimulates catabolism in C2C12 myotubes. American Journal of Physiology: Cell Physiology.

[34] Li Y.P., Schwartz R.J., Waddell I.D., Holloway B.R., Reid M.B. (1998). Skeletal muscle myocytes undergo protein loss and reactive oxygen-mediated NF-kappaB activation in response to tumor necrosis factor alpha. FASEB Journal.

[35] Lingwood D., Simons K. (2010). Lipid rafts as a membrane-organizing principle. Science.

[36] Livak K.J., Schmittgen T.D. (2001). Analysis of relative gene expression data using real-time quantitative PCR and the 2(-Delta Delta C(T)) method. Methods.

[37] Merrill A.H., Lingrell S., Wang E., Nikolova-Karakashian M., Vales T.R., Vance D.E. (1995). Sphingolipid biosynthesis de novo by rat hepatocytes in culture. Ceramide and sphingomyelin are associated with, but not required for, very low density lipoprotein secretion. Journal of Biological Chemistry.

[38] Milhas D., Clarke C.J., Idkowiak-Baldys J., Canals D., Hannun Y.A. (2010). Anterograde and retrograde transport of neutral sphingomyelinase-2 between the Golgi and the plasma membrane. Biochimica et Biophysica Acta.

[39] Min K., Smuder A.J., Kwon O.S., Kavazis A.N., Szeto H.H., Powers S.K. (2011). Mitochondrial-targeted antioxidants protect skeletal muscle against immobilization-induced muscle atrophy. Journal of Applied Physiology.

[40] Moncman C.L., Wang K. (1995). Nebulette: a 107 kD nebulin-like protein in cardiac muscle. Cell Motility and the Cytoskeleton.

[41] Moylan J.S., Smith J.D., Chambers M.A., McLoughlin T.J., Reid M.B. (2008). TNF induction of atrogin-1/MAFbx mRNA depends on Foxo4 expression but not AKT-Foxo1/3 signaling. American Journal of Physiology: Cell Physiology.

[42] Nikolova-Karakashian M., Karakashian A., Rutkute K. (2008). Role of neutral sphingomyelinases in aging and inflammation. Sub-Cellular Biochemistry.

[43] Nikolova-Karakashian M., Morgan E.T., Alexander C., Liotta D.C., Merrill A.H. (1997). Bimodal regulation of ceramidase by interleukin-1beta. Implications for the regulation of cytochrome P450 2C11. Journal of Biological Chemistry.

[44] Nikolova-Karakashian M.N., Rozenova K.A. (2010). Ceramide in stress response. Advances in Experimental Medicine and Biology.

[45] Okazaki T., Bell R.M., Hannun Y.A. (1989). Sphingomyelin turnover induced by vitamin D3 in HL-60 cells. Role in cell differentiation. Journal of Biological Chemistry.

[46] Olivera A., Buckley N.E., Spiegel S. (1992). Sphingomyelinase and cell-permeable ceramide analogs stimulate cellular proliferation in quiescent Swiss 3T3 fibroblasts. Journal of Biological Chemistry.

[47] Palomero J., Jackson M.J. (2010). Redox regulation in skeletal muscle during contractile activity and aging. Journal of Animal Science.

[48] Peraldi P., Hotamisligil G.S., Buurman W.A., White M.F., Spiegelman B.M. (1996). Tumor necrosis factor (TNF)-alpha inhibits insulin signaling through stimulation of the p55 TNF receptor and activation of sphingomyelinase. Journal of Biological Chemistry.

[49] Powers S.K., Hudson M.B., Nelson W.B., Talbert E.E., Min K., Szeto H.H., Kavazis A.N., Smuder A.J. (2011). Mitochondria-targeted antioxidants protect against mechanical ventilation-induced diaphragm weakness. Critical Care Medicine.

[50] Rath A., Glibowicka M., Nadeau V.G., Chen G., Deber C.M. (2009). Detergent binding explains anomalous SDS-PAGE migration of membrane proteins. Proceedings of the National Academy of Sciences of the United States of America.

[51] Reid M.B. (2008). Free radicals and muscle fatigue: of ROS, canaries, and the IOC. Free Radical Biology & Medicine.

[52] Reid M.B., Li Y.P. (2001). Cytokines and oxidative signalling in skeletal muscle. Acta Physiologica Scandinavica.

[53] Smith J.D., Moylan J.S., Hardin B.J., Chambers M.A., Estus S., Telling G.C., Reid M.B. (2011). Prion protein expression and functional importance in skeletal muscle. Antioxidants & Redox Signaling.

[54] Smith M.A., Reid M.B. (2006). Redox modulation of contractile function in respiratory and limb skeletal muscle. Respiratory Physiology & Neurobiology.

[55] Stoffel W., Jenke B., Blöck B., Zumbansen M., Koebke J. (2005). Neutral sphingomyelinase 2 (smpd3) in the control of postnatal growth and development. Proceedings of the National Academy of Sciences of the United States of America.

[56] Strle K., Broussard S.R., McCusker R.H., Shen W.H., LeCleir J.M., Johnson R.W., Freund G.G., Dantzer R., Kelley K.W. (2006). C-Jun N-terminal kinase mediates tumor necrosis factor-alpha suppression of differentiation in myoblasts. Endocrinology.

[57] Tabatadze N., Savonenko A., Song H., Bandaru V.V., Chu M., Haughey N.J. (2010). Inhibition of neutral sphingomyelinase-2 perturbs brain sphingolipid balance and spatial memory in mice. Journal of Neuroscience Research.

[58] Turinsky J., Nagel G.W., Elmendorf J.S., Damrau-Abney A., Smith T.R. (1996). Sphingomyelinase stimulates 2-deoxyglucose uptake by skeletal muscle. Biochemical Journal.

[59] Wu B.X., Clarke C.J., Hannun Y.A. (2010). Mammalian neutral sphingomyelinases: regulation and roles in cell signaling responses. Neuromolecular Medicine.

[60] Zheng T., Li W., Wang J., Altura B.T., Altura B.M. (2000). Effects of neutral sphingomyelinase on phenylephrine-induced vasoconstriction and Ca(2+) mobilization in rat aortic smooth muscle. European Journal of Pharmacology.

